# Establishment of novel in vitro culture system with the ability to reproduce oral biofilm formation on dental materials

**DOI:** 10.1038/s41598-021-00803-8

**Published:** 2021-10-27

**Authors:** Tomoki Kohno, Haruaki Kitagawa, Ririko Tsuboi, Yuma Nishimura, Satoshi Imazato

**Affiliations:** 1 Department of Advanced Functional Materials Science, Osaka University Graduate School of Dentistry, 1-8 Yamadaoka, Suita, Osaka 565-0871 Japan; 2 Department of Biomaterials Science, Osaka University Graduate School of Dentistry, 1-8 Yamadaoka, Suita, Osaka 565-0871 Japan

**Keywords:** Biofilms, Dental materials

## Abstract

Intensive research has been conducted with the aim of developing dental restorative/prosthetic materials with antibacterial and anti-biofilm effects that contribute to controlling bacterial infection in the oral cavity. In situ evaluations were performed to assess the clinical efficacy of these materials by exposing them to oral environments. However, it is difficult to recruit many participants to collect sufficient amount of data for scientific analysis. This study aimed to assemble an original flow-cell type bioreactor equipped with two flow routes and assess its usefulness by evaluating the ability to reproduce in situ oral biofilms formed on restorative materials. A drop of bacterial suspension collected from human saliva and 0.2% sucrose solution was introduced into the assembled bioreactor while maintaining the incubation conditions. The bioreactor was able to mimic the number of bacterial cells, live/dead bacterial volume, and volume fraction of live bacteria in the in situ oral biofilm formed on the surface of restorative materials. The usefulness of the established culture system was further validated by a clear demonstration of the anti-biofilm effects of a glass-ionomer cement incorporating zinc-releasing glasses when evaluated by this system.

## Introduction

In recent years, attempts have been made to confer bioactive properties to dental restorative/prosthetic materials^1,2^. Among the beneficial enhancements of dental materials with several bioactive properties, the development of restorative materials with antibacterial/anti-biofilm effects that contribute to controlling bacterial infection have been intensively researched. To assess the efficacy of these materials and their antibacterial and anti-biofilm properties in the oral cavity, in situ evaluations were performed by placing them in the oral cavity. Kreth et al., suggested that intraoral appliance biofilms should be used to evaluate the potential clinical efficacy of novel materials^3^. However, discomfort caused by wearing the appliance for a prolonged period of time deters people from participating in the study. Moreover, in situ assessment is not suitable for the evaluation of bioactive materials whose safety has not been sufficiently confirmed. Therefore, an in vitro culture system is required to reproduce oral biofilm formation on the surface of restorative materials and evaluate their antibacterial and anti-biofilm properties.

Several in vitro culture systems have been developed to simulate biofilms in diverse environments such as oral cavity and to evaluate biofilms themselves and the materials that serve as substrates for biofilm formation^4–6^. These in vitro culture systems are divided into two models: closed-system and open-system. The closed-system biofilm model is the most commonly used method, in which a material is immersed in a suspension of single-/mixed-species bacteria, followed by incubation for a certain period under static conditions. The experiments using the closed-system model are simple and highly reproducible; however, such model does not include an apparatus that can constantly supply nutrients essential for bacterial growth. Therefore, bacterial consumption of nutrients and accumulation of bacterial metabolites in the closed system leads to changes in culture conditions and bacterial growth and metabolism vary during the early and later stages of incubation^7^. This situation does not occur commonly in the oral cavity, and it can be interpreted that these systems are used to test the behavior of materials under extreme conditions, rather than to reproduce the oral cavity environment as closely as possible. However, this limitation can nevertheless make this model ideal for measuring the amount of active components leaking out of the material and concentrating on the supernatant broth, or their activity on the biofilms^6^.

The open-system biofilm model is a culture method in which bacterial suspensions and/or nutrients are constantly supplied to form a biofilm on the tested material. In these models, a flow of bacterial fluid or culture medium, using a peristaltic pump, can simulate salivary perfusion in the oral cavity. Robbins device or modified Robbins device (MRD) is utilized for the open-system model^8–11^. The MRD consists of a square channel pipe with equally spaced sampling ports attached to sampling plugs aligned with the inner surface, without disturbing the flow characteristics. This device can operate under different hydrodynamic conditions, from laminar to turbulent flow conditions^12^. Samples are placed in a pipe (i.e., a chamber) where bacterial fluid and nutrients are perfused; therefore, the specimens distal to the inlet experience a different nutritional environment than those proximal to the inlet due to consumption of the nutrients. The MRD was originally designed for low nutrient (drinking water, etc.) and high flow rate systems (that simulate the biofilms inside a water pipe, etc.), where this effect is less significant^7^. Contrarily, the environment to simulate salivary perfusion and reproduce an oral biofilm requires higher amount of nutrients at a lower flow rate. Furthermore, evaluation of the biofilm formed on materials which can release bioactive components (i.e., antimicrobials or ions, etc.) using the MRD system indicates that the released components are unintentionally incorporated into the bacterial fluid or culture medium in the chamber. Incorporation of antibacterial/anti-biofilm components has an influence on the growth of bacteria in the fluid and may inhibit the biofilm formed on the testing material distal to the inlet. This is the disadvantage of an open-system biofilm model with one large chamber, such as the MRD system.

Drip Flow Biofilm Reactor^®^ (DFR) (marketed by BioSurface Technologies, Bozeman, MT, USA) was developed to evaluate *Pseudomonas aeruginosa* biofilms^13^. This flow-cell type system includes separate chambers in which samples can be placed; thus, this bioreactor can be used to evaluate the releasing-type bioactive materials^14,15^. The DFR can evaluate up to six samples simultaneously, while a bacterial suspension is dripped onto each sample. Oral bacteria such as streptococci, lactobacilli, and actinomycetes catabolize carbohydrates as their main energy source^16^. Sucrose is considered the most cariogenic dietary carbohydrate since it is fermentable and serves as a substrate for the synthesis of extracellular polysaccharide (EPS), which is related to the attachment and maturation of the supra-gingival biofilm^17^. However, since DFR was not originally designed to reproduce oral biofilms, this device does not include a separate route for administering sucrose/glucose or other substances on the testing material other than the route for the bacterial suspension.

Here, we propose a flow-cell type bioreactor equipped with two flow routes that can supply sucrose as well as a bacterial suspension to reproduce biofilm formation similar to in the oral cavity. This study aimed to assemble a flow-cell type bioreactor equipped with two flow routes and assess its usefulness by evaluating the ability to reproduce in situ oral biofilms formed on a restorative material. Furthermore, the usefulness of the established culture system was validated by testing the anti-biofilm effects of glass-ionomer cements with the ability to release ions using the established in vitro evaluation system.

## Methods

### In situ evaluation of oral biofilm

Biofilm properties in the oral cavity were evaluated as a reference to biofilms’ behavior in the bioreactor to be constructed. Cured resin composites were used for in situ evaluation of oral biofilms. Resin composite paste (G-ænial Universal Flo, GC Corporation, Tokyo, Japan; hereafter denoted as GU) was filled in a mold (5 mm diameter, 1 mm thickness). The surface was covered with celluloid strips and a glass slide, and both sides were cured with a light activation unit (Alpha Light V, Morita, Kyoto, Japan) for a minute each. The light intensity on the turntable of this unit was 35 mW/cm^2^. The resin disc was stored for 24 h at 25 °C and then polished using silicon carbide grinding paper (Buehler, Lake Bluff, USA) from #120 to #1200. The sample was sterilized with ethylene oxide at 40 °C for 24 h. The disc was stored in distilled water for 24 h and high-performance liquid chromatography (Prominence series connected with SPD-20A UV–Vis detector, Shimadzu Corporation, Kyoto, Japan) confirmed the absence of release of unpolymerized monomers, which may affect biofilm formation (see Supplementary Fig. S1).

The in situ evaluation comprised five participants (three men and two women) aged between 20 and 38 years (mean 29.4 ± 6.5 years), who were students and staff at the Osaka University Graduate School of Dentistry. The participants did not demonstrate any clinical signs of caries, gingivitis, or periodontitis, and did not have history of any systemic diseases. The total number of decayed, missing, or filled teeth (DMF) in each participant was recorded as an index of dental caries, and the Community Periodontal Index (CPI) in each participant was recorded as an index of periodontal disease. A summary of the participant characteristics is presented in Table 1. The volunteers abstained from antibiotics for a period of 6 months before the study commenced. Written informed consent was obtained from all participants. The study design was reviewed and approved by the Ethics Committee of the Osaka University Graduate School of Dentistry and Osaka University Dental Hospital (approval number: R2-E19). The experiments were performed in accordance with the ethics guidelines for medical science studies of humans and the Declaration of Helsinki.

**Table 1:** Participant characteristics.

Participant number	Sex	Age	DMF	CPI
1	F	32	2	0
2	F	29	12	0
3	M	28	0	0
4	M	38	9	0
5	M	20	0	0

**Figure 1:**
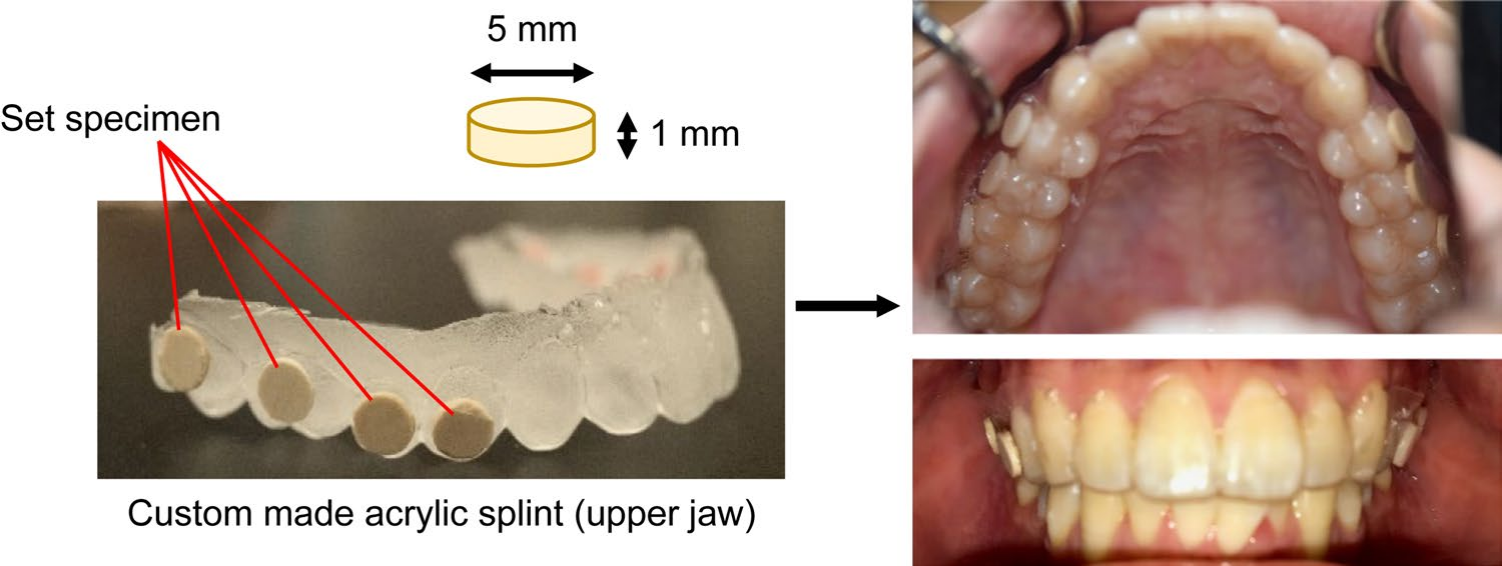
The custom-made acrylic splint used for in situ evaluation of oral biofilm.

The oral biofilms were evaluated using a modification of a previously reported in situ model^18^. The, participants wore a custom-made acrylic splint in their upper jaw for 24 h thus allowing formation of oral biofilms. The splint consisted of eight disc specimens, fixed with a cyanoacrylate-based glue (Aron Alpha, Toagosei Co., Ltd., Tokyo, Japan) in the region of the upper premolars and molars (Fig. 1). The participants wore the splint for 24 h, except for during meals and while brushing their teeth when the appliance was stored at > 95% humidity and 37 °C. After 24 h, the resin specimen was removed from the splint without disrupting the adherent biofilm. The specimen was gently irrigated twice with 1 mL phosphate-buffered saline (PBS; Wako, Osaka, Japan).

### Assembly of an original bioreactor for establishment of in vitro biofilm model

**Figure 2:**
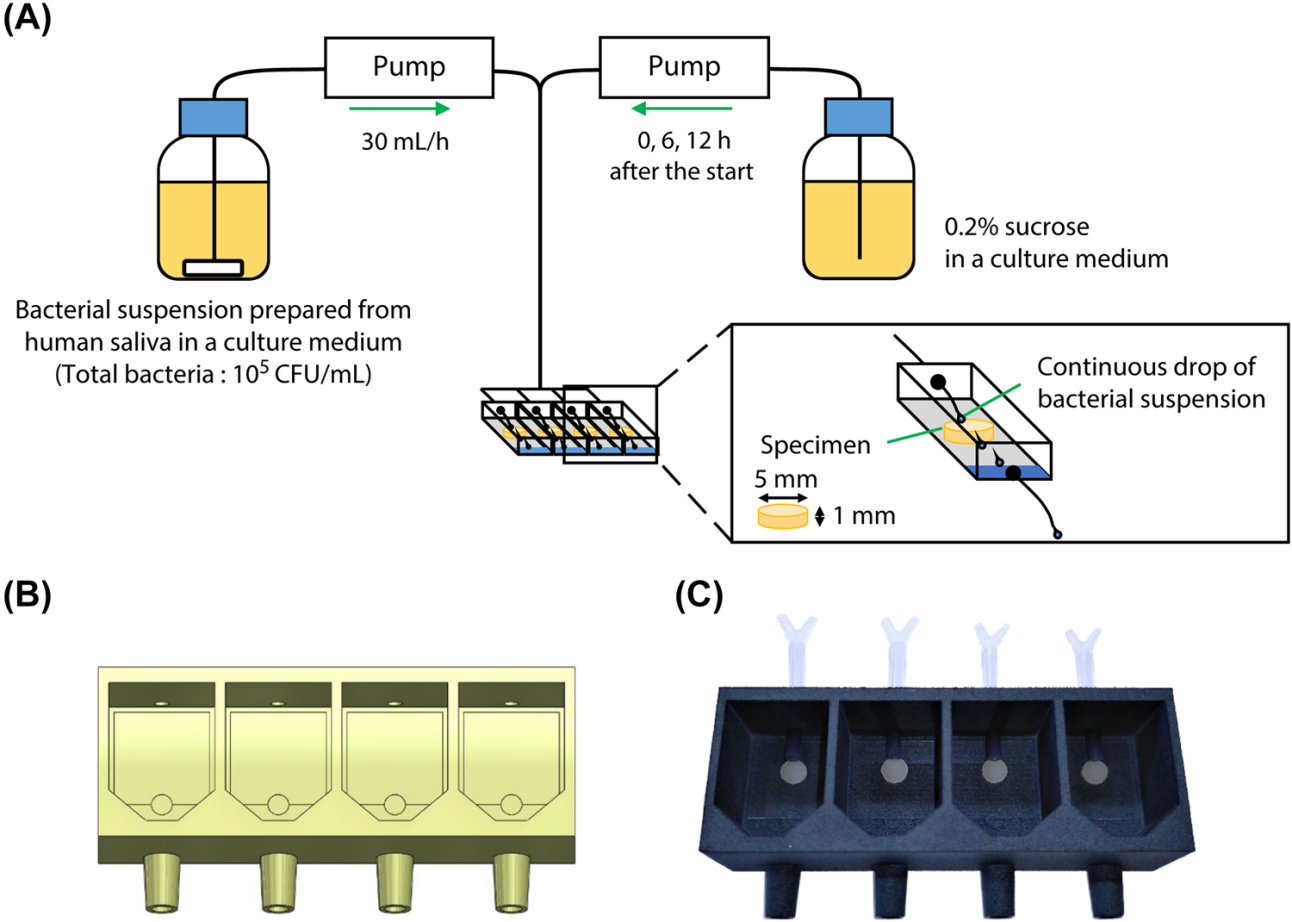
(**A**) Configuration of the original bioreactor assembled. (**B**) Design of flow chamber created using a 3D design software. (**C**) Appearance of the flow chamber fabricated using a 3D printer.

An original bioreactor was assembled, which aimed to simulate saliva flow conditions (Fig. 2A). The bacterial suspension in human saliva was flowed using peristaltic pumps (SJ-1211II-L, ATTO Corporation, Tokyo, Japan) on the disc-specimen set at a flow chamber. The flow chamber was designed using SolidWorks (Dassault Systèmes SE, Vélizy-Villacoublay, France) (Fig. 2B) and fabricated with a 3D printer (HP Jet Fusion 4200, HP Japan Inc., Tokyo, Japan) using a heat-resistant nylon (PA 12 GB, HP Japan Inc., Tokyo, Japan) (Fig. 2C). A lid that could seal the flow chamber in a hermetic fashion was made using the same 3D printer and material. Silicone tubes were connected to the upper two branches of the tube connector of the flow chamber and the solution bottles, and the mixed solution was dropped from the bottom of the silicone tube directly onto each sample fixed on the cover glass in the flow chamber. The whole assembled structure was sterilized through autoclaving before the experiment and set in an incubator (MCO-170AIC, Panasonic, Osaka, Japan) adjusted at 37 °C.

To prepare the bacterial suspension, human stimulated saliva was collected from five donors, who were the same volunteers as the ones for in situ evaluation. The donors did not brush teeth for 24 h and abstained from food and drink intake for 2 h prior to donating saliva. Stimulated saliva was collected during parafilm chewing and was kept on ice. An equal volume of saliva from each of the five donors was combined to form the saliva sample. The saliva was diluted in sterile glycerol to a saliva concentration of 70%, and stored at − 80 °C. The Ethics Review Committee of Osaka University Graduate School of Dentistry and Osaka University Dental Hospital approved the use of human saliva used for the incubation using the assembled bioreactor (Approval number: R1-E52).

### Assessment of incubation conditions using the assembled bioreactor

Unstimulated human saliva was collected from five donors as described above and filtered twice through a 0.22-µm syringe filter. The cured resin composite discs made using the same method as described above were immersed in 1 mL of filtered saliva for 2 h at 37 °C to form acquired salivary pellicle. The bacterial suspension used for the bioreactor was adjusted to approximately 10^5^ CFU/mL by diluting the collected saliva with brain heart infusion broth (BHI; Becton Dickinson, Sparks, MD, USA) or artificial saliva (AS; composition is specified in Table 2)^19^. Each bacterial suspension was dropped and flown at 30 mL/h onto the resin discs fixed in the flow chamber. After flowing the bacterial suspension for 0, 6, and 12 h, 0.2% sucrose solution was dropped three times on the discs through the second pump for 15, 30, and 60 min each (total 45, 90, and 180 min, respectively), which were abbreviated as sc45, sc90, and sc180, respectively. A group abbreviated as sc0 was the one, in which only the bacterial suspension (i.e. without the addition of 0.2% sucrose solution) was dropped. After incubation for 24 h, the resin disc was removed from the chamber and gently irrigated twice with 1 mL of PBS. The incubation conditions using the assembled bioreactor are listed in Table 3.

**Table 2:** Composition of the artificial saliva.

Component	Concentration (g/L)
Mucin (type II, porcine gastric)	2.5
Bacteriological peptone	2.0
Tryptone	2.0
Yeast extract	1.0
NaCl	0.35
KCl	0.2
CaCl_2_	0.2
Cysteine hydrochloride	0.1
Hemin	0.001
Vitamin K1	0.0002

**Table 3:** Incubation conditions in the assembled bioreactor.

Code	Culture medium	Addition time of 0.2% sucrose solution (min)
AS/sc180	AS	60 × 3 (total 180)
BHI/sc0	BHI	0
BHI/sc45	BHI	15 × 3 (total 45)
BHI/sc90	BHI	30 × 3 (total 90)
BHI/sc180	BHI	60 × 3 (total 180)

*AS* artificial saliva, *BHI* brain heart infusion broth.

### Analysis of biofilms formed on the specimens

To quantify bacterial cells in biofilms, biofilms that formed at the surface of the resin discs were scraped using a microbrush. The microbrush was transferred to 10 mL of PBS and sonicated for 10 min in an ultrasonic bath operating at 37 kHz and 300 W, to detach the bacteria. The suspension was serially diluted, and aliquots of the suspension were spread on trypticase soy agar with 5% sheep blood (Nippon Becton Dickinson, Tokyo, Japan). The number of colonies was counted after anaerobic incubation at 37 °C for 24 h.

Biofilms formed on the samples were stained using LIVE/DEAD^®^ BacLight™ bacterial viability kits (L7007, Molecular Probes, Eugene, OR, USA) for observation using confocal laser scanning microscopy (CLSM). Staining was carried out according to the manufacturer’s instructions; 2 µL of component A (1.67 mM SYTO^®^ 9 dye and 1.67 mM propidium iodide in dimethyl sulfoxide) and 2 µL of component B (1.67 mM SYTO^®^ 9 dye and 18.3 mM propidium iodide in dimethyl sulfoxide) were mixed in 1 mL of distilled water. The mixed solution (100 µL) was dropped onto the resin disc and incubated at 37 °C for 15 min in the dark. After gentle irrigation with distilled water, the discs were visualized using a CLSM (LSM 700, Carl Zeiss, Oberkochen, Germany) at 488 and 555 nm for excitation, and 500 and 635 nm for emission. Images were obtained using ZEN Imaging Software (Carl Zeiss, Oberkochen, Germany). A preliminary image was acquired to determine the acquisition parameters, and the settings were kept constant for all images. Images were acquired at 12-bit depth with a resolution of 1024 × 1024 pixels with the following settings: objective = Plan-Apochromat 10 × /0.45 M27; speed = 8; pinhole size = 34 μm; digital offset = 0; master gain (Ch1, SYTO^®^ 9) = 493; master gain (Ch2, propidium iodide) = 706; z-stack interval = 3 μm. Three images were obtained from one sample, and the images were analyzed using Imaris software (Bitplane, Zurich, Switzerland) to determine the volume of bacteria with intact cell membranes or damaged cell membranes. These values were used to calculate the volume fraction of bacteria with intact cell membranes.

### Evaluation of anti-biofilm effect of glass-ionomer cements (GICs) using the assembled bioreactor

A conventional fluoride-releasing GIC (Fuji VII, GC Corporation, Tokyo, Japan; hereafter denoted as F7) and a GIC with the ability to release zinc, calcium, and fluoride ions (Caredyne^®^ Restore, GC Corporation, Tokyo, Japan; hereafter denoted as CA) were used to evaluate the anti-biofilm effect using the assembled bioreactor. The powder and liquid for each GIC were mixed in a ratio of 1.8:1 (w/w) for F7 or 2.3:1 (w/w) for CA. The paste was poured into a mold (5 mm diameter, 1 mm thickness), the surface covered with a celluloid strip and glass slide and stored at 25 °C for 24 h. The set GIC structures were polished using silicon carbide grinding papers (#120 to #1200; Buehler, Lake Bluff, USA).

The bacterial suspension was dropped and flowed at 30 mL/h onto each GIC sample fixed in the flow chamber. After flowing the bacterial suspension for 0, 6, and 12 h, a 0.2% sucrose solution was dropped on the specimen three times for 15 min each (total 45 min). After incubation for 24 h, the samples gently irrigated twice with 1 mL of PBS. The biofilm formed on the sample was analyzed by colony counts and CLSM observations using the method described in the previous section. The resin composites (GU) were used as controls.

### Statistical analysis

Statistical analyses were performed using Statistical Package for the Social Sciences Statistics 25 (IBM Corp., Armonk, NY, USA). Homogeneity of variance was initially confirmed. The results for quantification of bacterial cells in biofilms were statistically analyzed using analysis of variance (ANOVA) and Tukey’s honest significant difference (HSD) test. The volume of bacteria with intact cell membranes and damaged cell membranes was statistically analyzed by ANOVA and Dunnett’s T3 test. The volume fraction of bacteria with intact cell membranes was analyzed by ANOVA and Dunnett’s T3 test after logit conversion. *p* values below 0.05 were considered to indicate statistical significance.

## Results

### In situ evaluation of oral biofilm

**Figure 3:**
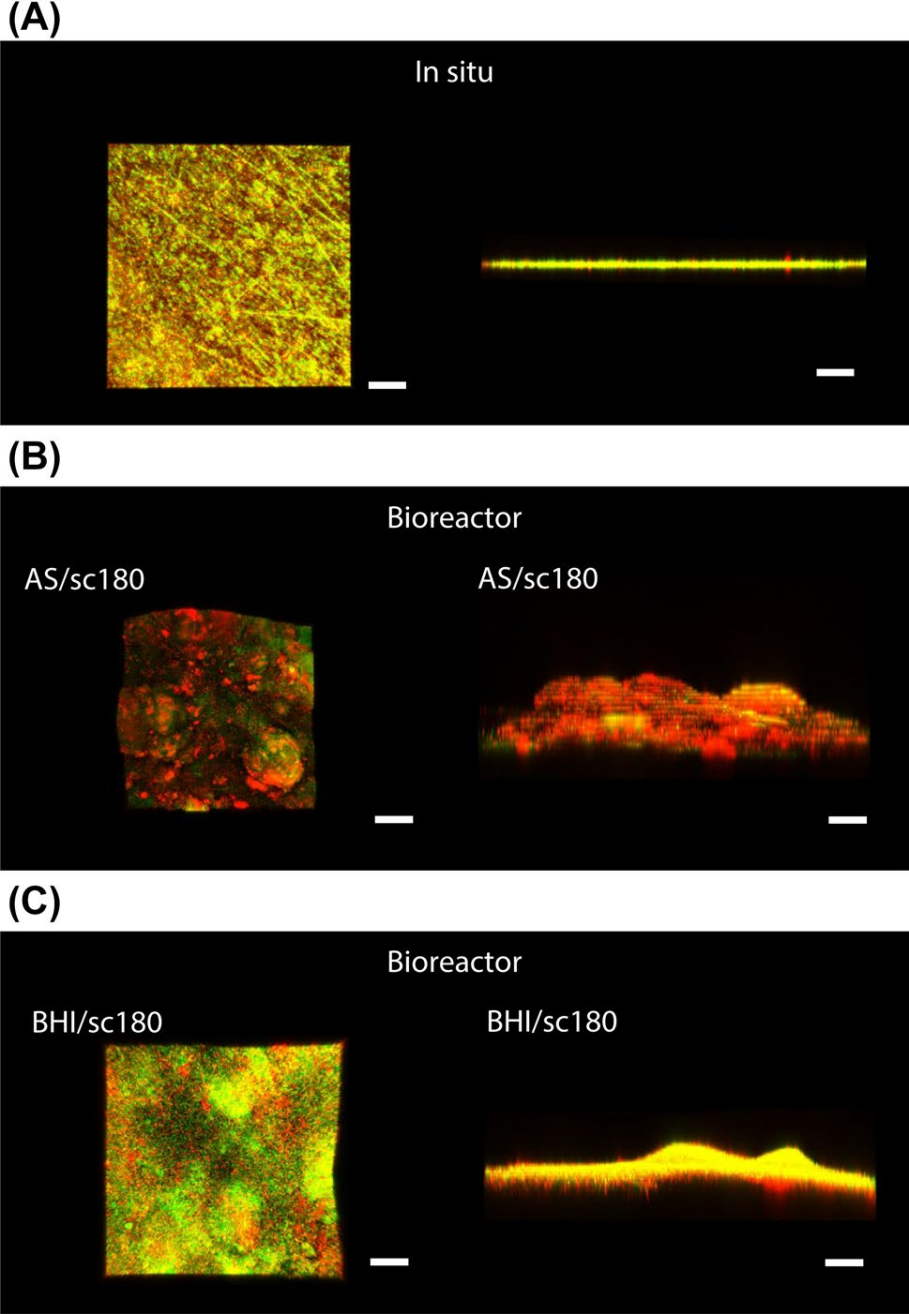
Representative surface and cross-sectional confocal laser scanning microscope images of biofilm formed on the resin composites. (**A**) in situ biofilm. (**B**,**C**) Biofilms formed after incubation with artificial saliva in the assembled bioreactor at AS/sc180 (**B**) and BHI/sc180 (**C**). AS, artificial saliva. BHI, Brain heart infusion broth. Scale bar 100 μm.

Representative surface and cross-sectional CLSM images of the in situ biofilms formed on the resin composites are shown in Fig. 3A. The number of bacterial cells in the in situ biofilm formed on the resin composite disc was 2.2 × 10^7^ ± 1.9 × 10^7^ CFU. The volume of bacteria with intact cell membranes and damaged cell membranes were determined using Imaris imaging software as 1.92 × 10^6^ ± 0.93 × 10^6^ μm^3^ and 2.80 × 10^6^ ± 0.96 × 10^6^ μm^3^, respectively. The volume fraction of bacteria with intact cell membranes was 40.7 ± 15.5%.

### Assessment of incubation conditions using the assembled bioreactor

**Figure 4:**
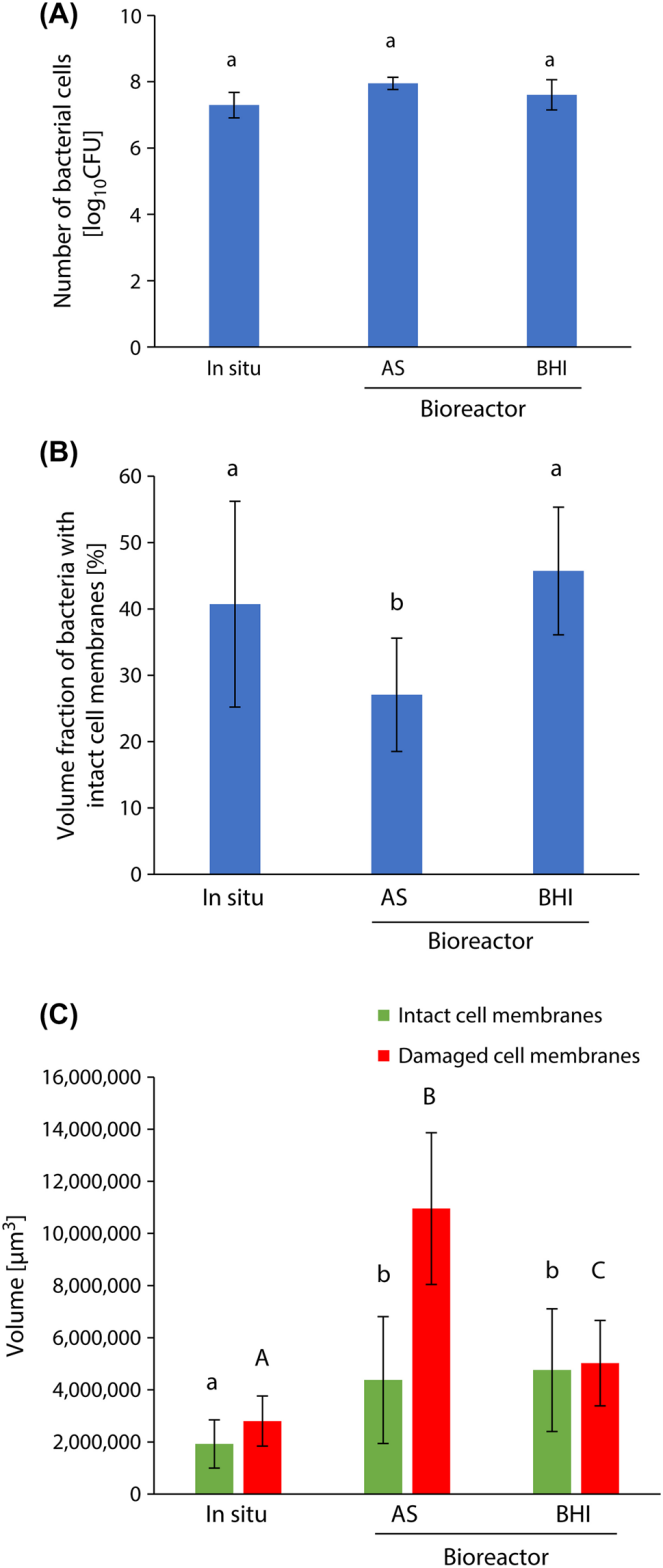
Comparison between properties of biofilms formed using different media in the assembled bioreactor. (**A**) The number of bacterial cells in the biofilm. a: No significant difference is observed between bars labeled with the same letter (analysis of variance, Tukey's honest significant difference test, *p* > 0.05, n = 5). (**B**) The volume fraction of bacteria with intact cell membranes. a, b: No significant difference is observed between bars labeled with the same letter (analysis of variance, Dunnett T3 test, *p* > 0.05, n = 10). (**C**) The volume of bacteria with intact cell membranes and damaged cell membranes. a, b, A, B, C: No significant difference is observed between bars labeled with the same letter (analysis of variance, Dunnett T3 test, *p* > 0.05, n = 10). Error bars represent the standard deviation.

Figure 3B,C show the surface and cross-sectional CLSM images of biofilms formed on the resin composites after incubation using the assembled bioreactor under the conditions of AS/sc180 and BHI/sc180, respectively. No significant difference in the number of bacterial cells was observed between the in situ biofilm and the AS/sc180 and BHI/sc180 groups (*p* > 0.05, ANOVA, Tukey’s HSD test, n = 5) (Fig. 4A). The volume fraction of bacteria with intact cell membranes for group AS/sc180 was determined as 27.9 ± 8.8% and was significantly smaller than that in the in situ biofilm (*p* < 0.05, ANOVA, Dunnett’s T3 test, n = 10). The volume fraction of bacteria with intact cell membranes of group BHI/sc180 was 47.3 ± 7.3%, which was not significantly different from that of the in situ biofilm (*p* > 0.05, ANOVA, Dunnett’s T3 test, n = 10) (Fig. 4B). The volume of bacteria with intact cell membranes and damaged cell membranes in both AS/sc180 and BHI/sc180 groups were significantly greater than that of the in situ biofilm (*p* < 0.05, ANOVA, Dunnett’s T3 test, n = 10) (Fig. 4C). Based on these results, BHI broth was used as a medium to prepare the bacterial suspension for subsequent evaluation.

**Figure 5:**
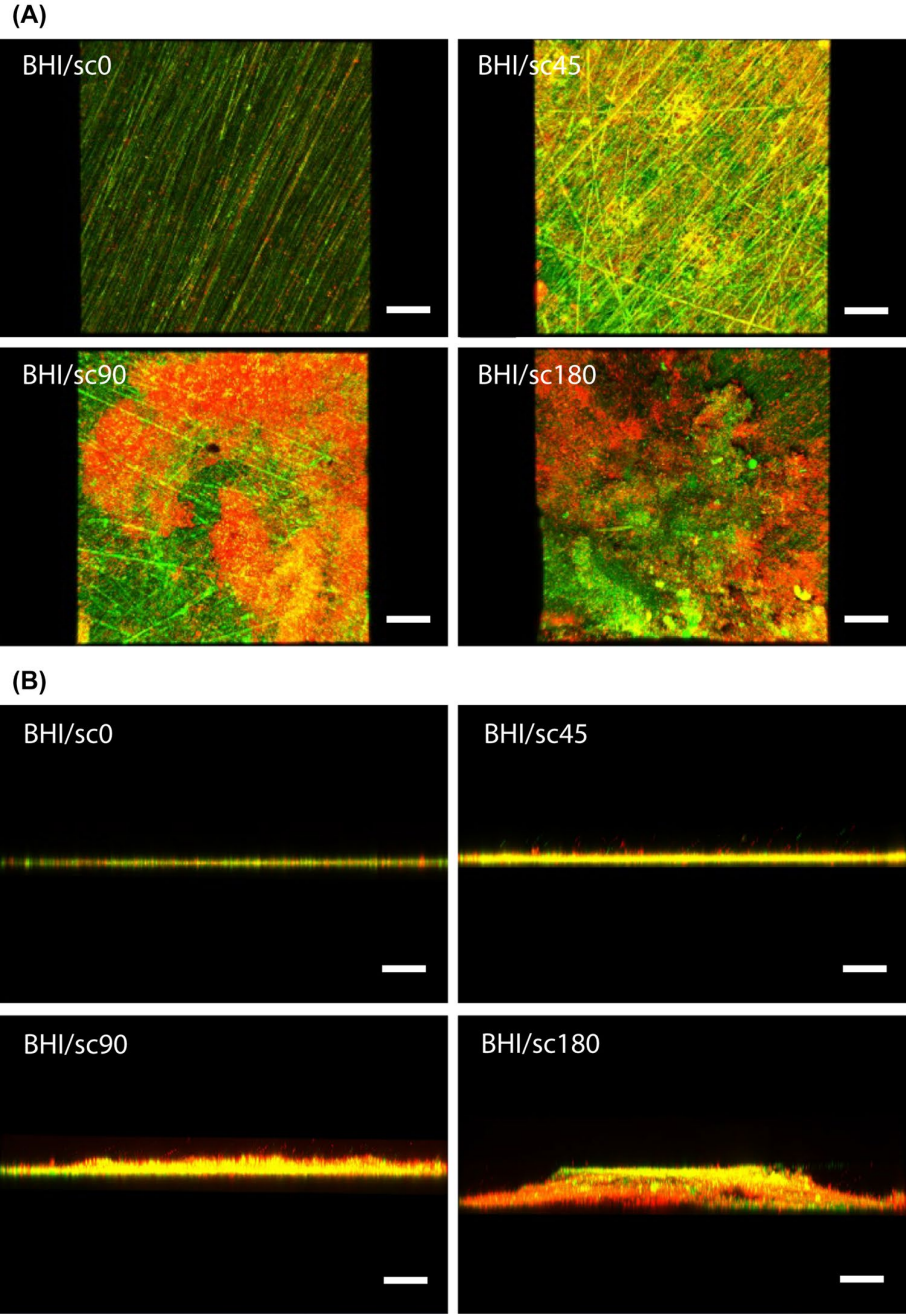
Representative (**A**) surface and (**B**) cross-sectional confocal laser scanning microscope images of biofilms formed on resin composites after incubation using the assembled bioreactor under the condition of the addition of 0.2% sucrose at different times. Scale bar 100 μm.

**Figure 6:**
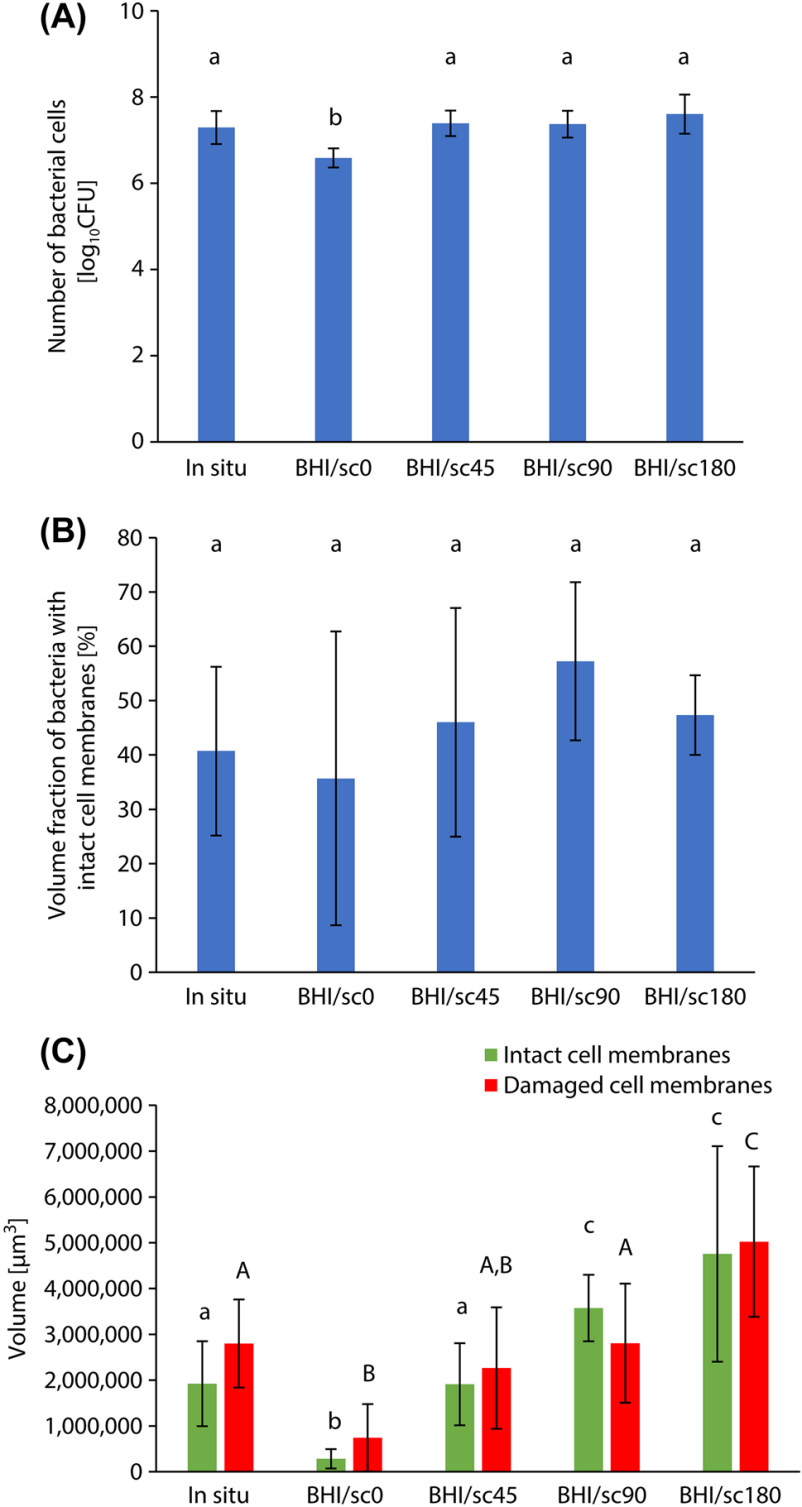
Comparison of properties of biofilms formed after incubation using the assembled bioreactor under different sucrose addition times. (**A**) The number of bacterial cells in the biofilm. a, b: No significant difference is observed between bars labeled with the same letter (analysis of variance, Tukey's honest significant difference test, *p* > 0.05, n = 5). (**B**) The volume fraction of bacteria with intact cell membranes. a: No significant difference is observed between bars labeled with the same letter (analysis of variance, Dunnett T3 test, *p* > 0.05, n = 10). (**C**) The volume of bacteria with intact cell membranes and damaged cell membranes. a, b, c, A, B, C: No significant difference is observed between bars labeled with the same letter (analysis of variance, Dunnett T3 test, *p* > 0.05, n = 10). Error bars represent the standard deviation.

Figure 5A,B show the surface and cross-sectional CLSM images of biofilms formed on the resin composite discs after incubation under the condition in which sucrose was added at different times. The number of bacterial cells in the biofilm of the BHI/sc0 group was significantly smaller than that of the in situ biofilm (*p* < 0.05, ANOVA, Tukey’s HSD test, n = 5). No significant difference in the number of bacterial cells was observed between the in situ biofilm and BHI/sc45, BHI/sc90, and BHI/sc180 groups (*p* > 0.05, ANOVA, Dunnett’s T3 test, n = 10) (Fig. 6A). In addition, the volume fraction of bacteria with intact cell membranes formed by the incubation conditions of BHI/sc0, BHI/sc45, BHI/sc90, and BHI/sc180 were not significantly different from those in the biofilm formed in the oral cavity (*p* > 0.05, ANOVA, Dunnett’s T3 test, n = 10) (Fig. 6B). In contrast, the volume of bacteria with intact cell membranes and damaged cell membranes was increased with increasing sucrose addition time. No significant difference in both the volume of bacteria with intact cell membranes and damaged cell membranes was observed between the in situ biofilm and BHI/sc45 group (*p* > 0.05, ANOVA, Dunnett’s T3 test, n = 10) (Fig. 6C).

### Evaluation of anti-biofilm effect of GICs using the assembled bioreactor

**Figure 7:**
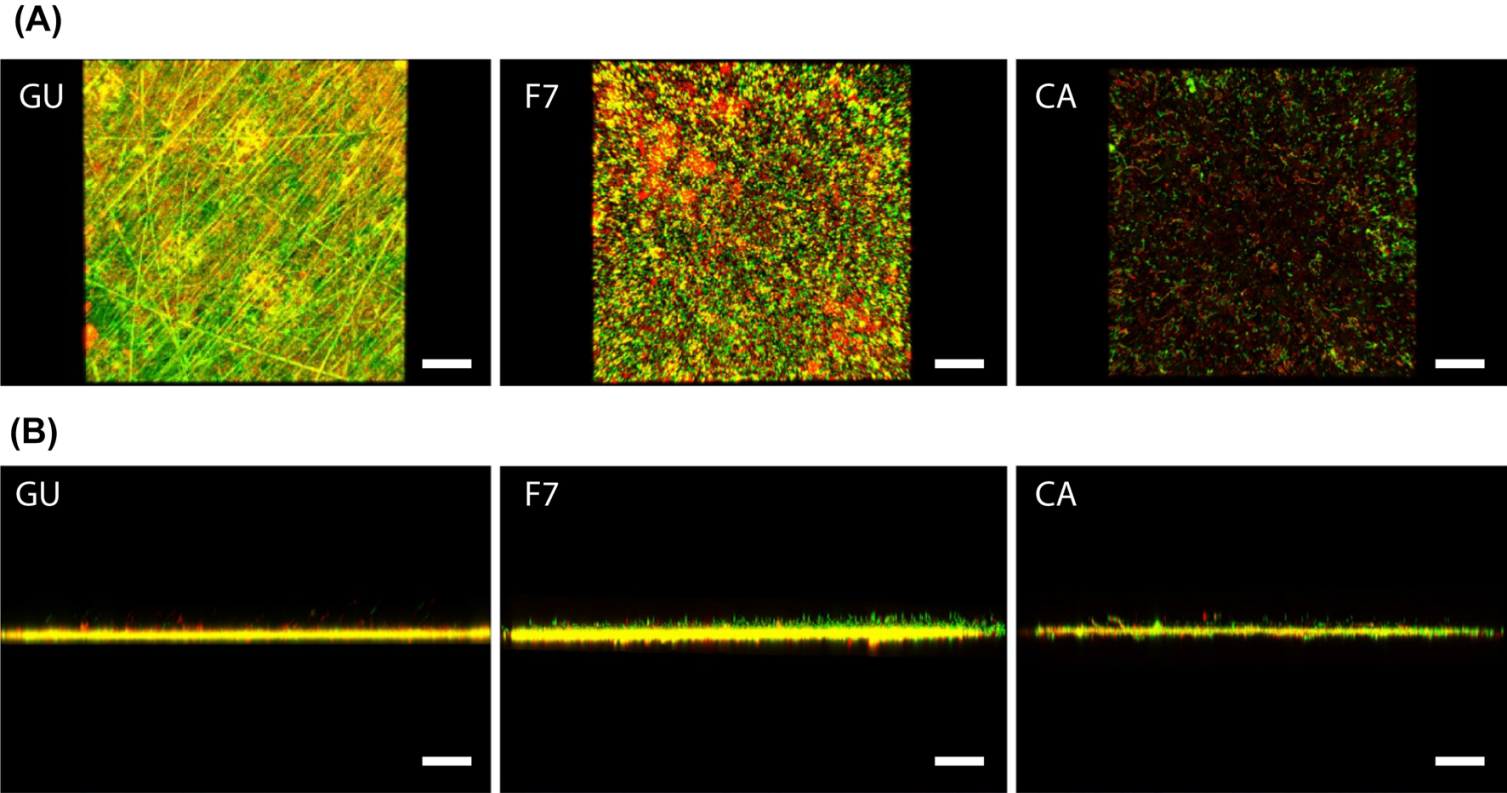
Representative (**A**) surface and (**B**) cross-sectional confocal laser scanning microscope images of biofilm formed on the specimens of GU, F7, and CA specimens. Scale bar, 100 μm.

**Figure 8:**
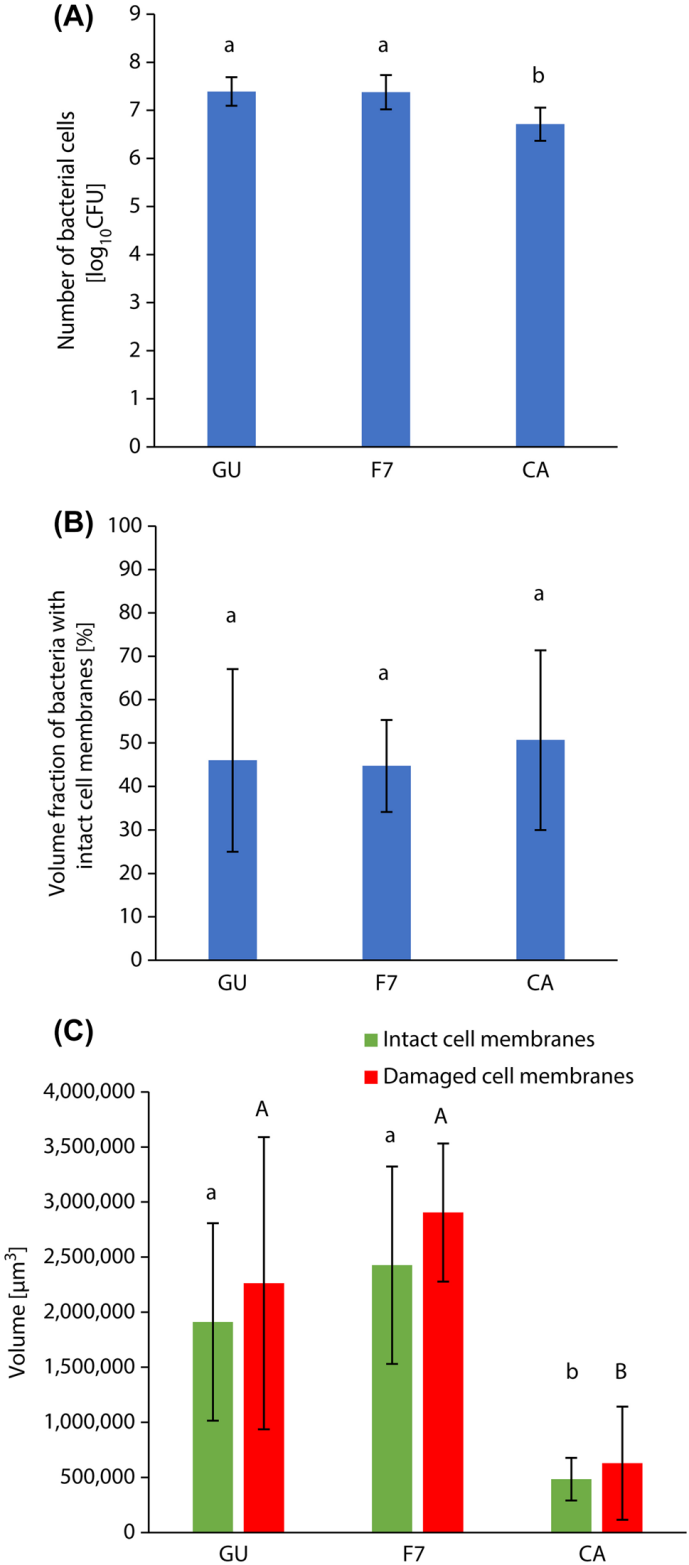
Comparison between properties of biofilms formed on different restorative materials in the assembled bioreactor. (**A**) The number of bacterial cells in the biofilm. a, b: No significant difference is observed between bars labeled with the same letter (analysis of variance, Tukey's honest significant difference test, *p* > 0.05, n = 5). (**B**) The volume fraction of bacteria with intact cell membranes. a: No significant difference is observed between bars labeled with the same letter (analysis of variance, Dunnett T3 test, *p* > 0.05, n = 10). (**C**) The volume of bacteria with intact cell membranes and damaged cell membranes. a, b, A, B: No significant difference is observed between bars labeled with the same letter (analysis of variance, Dunnett T3 test, *p* > 0.05, n = 10). Error bars represent the standard deviation.

Figure 7A,B show the surface and cross-sectional images of biofilms formed on the samples of GU, F7, and CA. The number of bacterial cells in the biofilm in the CA group was significantly smaller than that in the GU and F7 biofilms (*p* < 0.05, ANOVA, Tukey’s HSD test, n = 5) (Fig. 8A). There was no significant difference in the volume fraction of bacteria with intact cell membranes formed in all the groups. (*p* > 0.05, ANOVA, Dunnett’s T3 test, n = 10) (Fig. 8B), whereas the volume of bacteria with intact cell membranes and damaged cell membranes in the biofilm in the CA group were significantly smaller than those of the GU and F7 biofilms (*p* < 0.05, ANOVA, Dunnett’s T3 test, n = 10) (Fig. 8C).

## Discussion

Understanding the characteristics of oral biofilms is crucial for developing methods to effectively control biofilms, thereby preventing oral diseases such as dental caries and periodontal disease. Several in situ evaluations have been performed to investigate oral biofilm formation on the surface of teeth or dental materials^20–22^. In the present study, we used an in situ model in which the participants wore a custom-made acrylic splint, with discs fixed in the region of the upper premolars and molars. Commercialized resin composites were fixed on the acrylic sprint as these are bio-inert materials. The number of bacterial cells in the biofilm on the resin composite disc, after being exposed to the oral environment for 24 h, was approximately 2 × 10^7^ CFU, similar to the results previously reported for in situ biofilms formed on hydroxyapatite discs^23^. In contrast, the volume fraction of bacteria with intact cells in the biofilm formed on resin composites was only 40.7%, which was smaller than that on the hydroxyapatite discs^19^. Auschill et al., reported that the vitality of bacteria in in situ biofilms formed on resin composites was between 4 and 21%, which was smaller than that on inorganic ceramic materials (34–86%)^24^. This tendency was consistent with our result as the ratio of intact bacteria on resin composites was smaller than that (as previously reported) on inorganic hydroxyapatite.

In situ evaluation allows the apparatus to be removed during meals and tooth brushing, minimizing the physical and mental stresses associated with placing the tested material in oral cavities. Nevertheless, the discomfort caused by wearing it for a prolonged period deters volunteers from participating. Thus, not enough participants are available to prove the effectiveness of each material. Therefore, to establish an in vitro evaluation system that can reproduce in situ oral biofilms formed on restorative materials, a new bioreactor was developed in the present study. AS, a chemically defined medium, is used in open-system biofilm models to simulate salivary flow^20,25^. The composition of AS is adjusted to grow oral bacteria; however, use of AS decreases the volume fraction of bacteria with intact cell membranes in the biofilm after incubation using the saliva-derived bacterial suspension compared with that in the in situ biofilm. In contrast, BHI, a nutrient-rich medium can be used to culture a variety of microorganisms, including streptococci, pneumococci, and meningococci^26–28^. BHI is made by combining an infusion from boiled bovine or porcine heart and brain with a variety of other nutrients^29^. Our results suggest that the use of BHI medium, which is richer in peptides and amino acids than AS, increased the viability of bacteria in the biofilm. Furthermore, the volume fraction of bacteria with intact cell membranes was the same as that in the in situ biofilm. Based on these results, it was found that BHI medium was suitable for reproducing in situ oral biofilms and preparing the saliva-derived bacterial suspension in our in vitro system using the assembled bioreactor.

Sucrose is a substrate for the synthesis of EPS, which promotes bacterial adherence to the tooth surface and contributes to structural integrity of oral biofilms^30–33^. In this study, biofilm formation on resin composites was evaluated under conditions of sucrose addition for different length of time. The results indicated that increasing the time of sucrose addition increased the volume of bacteria in the biofilms. EPS increases the bulk and porosity of the dental plaque matrix, thereby allowing more substrate to diffuse to the surface^17^. The results of this study indicate that volume of the biofilm can be adjusted by controlling the amount of sucrose added. Although diverse analyses of biofilm characteristics such as microbial composition and total amount of EPS in the biofilm are required, this study demonstrated that biofilms with properties similar to those of in situ biofilms could be formed on resin composites by adding 0.2% sucrose solution for a total of 45 min.

This in vitro culture system can accurately evaluate the antibacterial/anti-biofilm effects of the antimicrobial-releasing-type materials, since it includes separate chambers in which each sample can be placed. In this study, we selected a conventional fluoride-releasing GIC and a recently developed multiple-ion-releasing GIC to validate whether the established in vitro culture system can be utilized for evaluating the efficacy of antimicrobial-releasing-type restorative materials. GICs were developed in the 1970s and have been widely used as restorative materials and luting cements because of their performance under wet conditions^34^. GICs are capable of releasing fluoride, which makes teeth caries resistant by formation of fluorapatite crystals and remineralization of damaged enamel and dentin^35,36^. Fluoride released from GICs has the potential to reduce the number of bacteria or interfere with bacterial metabolism in dental plaque^37,38^, but the amount of fluoride released from GICs is insufficient and short-lived, thus ineffective in inhibiting bacterial growth^36^. BioUnion filler was developed as a glass powder composed of SiO_2_, ZnO, CaO, and F, and can be categorized as a bio-functional multi-ion-releasing filler^2^. It has a silicon-based glass structure and is capable of releasing Zn^2+^, Ca^2+^, and F^−^. Zn^2+^ is known to exhibit antibacterial effects against oral bacteria, and its minimum inhibitory concentration and minimum bactericidal concentration values against *Streptococcus mutans* are lower than those of fluoride^39,40^. Liu et al., reported that the release of Zn^2+^ from the BioUnion filler was accelerated under acidic conditions^40^. Such technology enables the on-demand release of antimicrobial components from materials. Once dental plaque is formed on the surface and acidogenic bacteria produce acids, a greater amount of Zn^2+^ is released and effectively attacks the cariogenic bacteria in the plaque. The GIC containing BioUnion filler for root surface restoration (Caredyne^®^ Restore) is currently in the market. The closed-system biofilm model (i.e., incubation under static conditions) was used to demonstrate that acidity-induced release of Zn^2+^ from the GIC containing BioUnion filler effectively inhibited the growth and adherence of *Streptococcus mutans*, *S. sobrinus, S. oralis, S. mitis*, *Actinomyces naeslundii*, and *Fusobacterium nucleatum*^41^. Previously, we developed a saliva-drop setting assembly that can flow AS at 32 mL/h and drop acetate buffer solution (pH 4.5) three times per day to simulate in vivo conditions of the oral cavity, to investigate the ion releasing properties of BioUnion filler-containing and conventional fluoride-releasing GICs^42^. GIC containing BioUnion filler released Zn^2+^ and F^−^ under acidic conditions. During repeated exposure to acid for 7 days while flowing the AS, the concentrations of Zn^2+^ could be maintained at a level that inhibited *S. mutans* and multi-species biofilm formation. In contrast, the concentration of F^−^ released from both BioUnion filler-containing and conventional fluoride-releasing GICs was not sufficient to inhibit biofilm formation.

Results of this study revealed that GIC containing BioUnion filler reduced the volume of biofilm formed as well as the number of bacteria in the biofilm. It has been reported that the pH values of several oral bacterial species (i.e., *S. mutans*, *S. sobrinus*, *S. oralis*, *S. mitis*, *A. naeslundii*, and *F. nucleatum*) related to dental plaque formation decreased (4.2–4.7) when they were cultured under sucrose-supplemented media^41,43,44^. Sucrose also increases the porosity of the biofilm, allowing carbohydrates to diffuse into the deepest parts of the biofilm, which results in low plaque pH values due to microbial catabolism^17^. Therefore, in the present study, it was suggested that Zn^2+^ could be released by pH reduction in the bacterial suspension with the addition of 0.2% sucrose solution for a total of 45 min, leading to the inhibition of oral biofilm formation on its surface. Contrarily, no significant difference in the number of bacterial cells in the biofilm and the volume of the biofilm was found between conventional fluoride-releasing GIC and resin composites. Therefore, the results obtained in this study were comparable to those previously reported by simple in vitro testing demonstrating that BioUnion filler-containing GICs inhibit the growth and adherence of oral bacteria more effectively than conventional fluoride-releasing GICs^41,42^. Based on these findings, the in vitro culture system established in the present study is suitable for evaluating the efficacy of restorative materials with antibacterial and anti-biofilm effects. However, further improvements that can monitor the concentration of active ingredients released from the restorative/prosthetic materials will help understand the mechanism of antibacterial and anti-biofilm properties.

In conclusion, we assembled an original bioreactor and reported that a biofilm, similar to those formed on resin composites in the oral cavity, could be formed using a bacterial suspension prepared from human saliva in BHI medium in the presence of 0.2% sucrose solution added for a total of 45 min. Furthermore, the usefulness of the established culture system was validated by a clear demonstration of the anti-biofilm effects of a glass-ionomer cement incorporating zinc-releasing glasses when evaluated by this system.

## Supplementary Information

Supplementary Information.

## Data Availability

The datasets used and analyzed during the current study are available from the corresponding author on reasonable request.
