# Supplementary material for: Establishment of novel in vitro culture system with the ability to reproduce oral biofilm formation on dental materials
Source: Sci Rep. 2021 Oct 27;11:21188. doi: 10.1038/s41598-021-00803-8 (PMC8551238; doi:10.1038/s41598-021-00803-8)
Supplement: Supplementary file 1 — Supplementary Information. [file 41598_2021_803_MOESM1_ESM.docx]

**Supplementary Information: Establishment of novel *in vitro* culture system with the ability to reproduce oral biofilm formation on dental materials**

Tomoki Kohno^a^, Haruaki Kitagawa^b^, Ririko Tsuboi^a^, Yuma Nishimura^b^, Satoshi Imazato^a,b,^*****

^a^Department of Advanced Functional Materials Science, Osaka University Graduate School of Dentistry, 1-8 Yamadaoka, Suita, Osaka 565-0871, Japan

^b^Department of Biomaterials Science, Osaka University Graduate School of Dentistry, 1-8 Yamadaoka, Suita, Osaka 565-0871, Japan

***Corresponding Author:**

Satoshi Imazato

1-8 Yamadaoka, Suita, Osaka 565-0871, Japan

Tel.: +81-(0)6-6879-2915

Fax: +81-(0)6-6879-2916

E-mail address: imazato@dent.osaka-u.ac.jp

**Supplementary Fig S1. Chromatogram of resin composites cured disc, uncured paste and control.**

Four cured disc specimens (5 mm diameter, 1 mm thickness) of resin composites (G-ænial Universal Flo, GC corporation, Tokyo, Japan) and same weight of uncured resin composites paste were immersed in the 10 mL of Milli-Q water for 24 hrs and the eluate was analyzed by HPLC (series connected with SPD-20A UV-Vis detector,). Milli-Q water was used as the control. The detection was performed at 254 nm on a Puresil C18 Column (4.6 mm × 250 mm, Waters, Milford, MA, USA) using a Prominence system (Shimadzu corporation, Kyoto, Japan). The column was maintained at 28 °C and the samples were eluted for 20 min. The mobile phase consisted of acetonitrile and 0.02% phosphoric acid with 100 mM sodium perchlorate. The volume of sample injected was 10 μL.
